# Strong increase of leukocyte apha‐galactosidase A activity in two male patients with Fabry disease following oral chaperone therapy

**DOI:** 10.1002/mgg3.894

**Published:** 2019-08-08

**Authors:** Foudil Lamari, Wladimir Mauhin, Fairouz Koraichi, Walid Khrouf, Celine Bordet, Jonathan London, Olivier Lidove, Philippe Charron

**Affiliations:** ^1^ Laboratoire de Biochimie Métabolique APHP, Hôpitaux Universitaires Pitié‐Salpêtrière Paris France; ^2^ GRC 2011‐Neurométabolisme Université Pierre et Marie‐Curie‐Sorbonne Paris France; ^3^ Service de Médecine Interne, Centre de Référence des Maladies Lysosomales Groupe Hospitalier Diaconesses‐Croix St Simon Paris France; ^4^ Centre de Référence pour les Maladies Cardiaques Héréditaires, Département de Génétique APHP, Hôpital Pitié‐Salpêtrière Paris France; ^5^ INSERM, UMR_S 1166 and ICAN Institute for Cardiometabolism and Nutrition Sorbonne Université Paris France

**Keywords:** alpha‐galactosidase A, cardiomyopathy, Fabry disease, leukocytes, migalastat

## Abstract

**Background:**

Fabry disease (OMIM 301500) is an X‐linked disorder caused by alpha‐galactosidase A (α‐Gal A) deficiency. The administration of a pharmacologic chaperone (migalastat) in Fabry patients with amenable mutations has been reported to improve or stabilize organ damages and reduce *lyso*‐Gb3 plasma level. An increase of α‐Gal A activity has been observed in vitro in cells expressing amenable *GLA* mutations when incubated with migalastat. The impact of the drug on α‐Gal A in vivo activity has been poorly studied.

**Methods:**

We conducted a retrospective analysis of two unrelated male Fabry patients with p.Asn215Ser (p.N215S) variant.

**Results:**

We report the important increase of α‐Gal A activity in blood leukocytes reaching normal ranges of activity after about 1 year of treatment with migalastat. Cardiac parameters improved or stabilized with the treatment.

**Conclusion:**

We confirm in vivo the effects of migalastat that have been observed in N215S carriers in vitro. The increase of α‐Gal A activity may be the strongest marker for biochemical efficacy. The normalization of enzyme activity could become the new therapeutic target to achieve.

## 1 INTRODUCTION

Fabry disease (FD) is an X‐linked disorder caused by mutations in *GLA*, coding for α‐galactosidase A enzyme (α‐Gal A). FD is pan‐ethnic and has an incidence of 1 per 100,000, which is probably underestimated (Germain, 2010). The α‐Gal A enzyme deficiency causes multi‐organ deposition of its main substrate, globotriaosylceramide (Gb3). The clinical presentation of FD is heterogeneous, ranging from an isolated cardiomyopathy to a systemic disease with acroparesthesia, deafness, cornea verticillata, abdominal pain, progressive renal failure, and cerebral stroke with poor outcome (Germain, 2010). Left ventricular (LV) myocardial hypertrophy (LVMH) and cardiac fibrosis have been suggested as risk factors for arrhythmias and sudden death (Krämer et al., 2014). Currently, it is widely accepted that enzyme replacement therapy (ERT) provides benefits in terms of cardiac hypertrophy and renal disease, at least when initiated in the early stage of the disease (El Dib et al., 2017). Migalastat (or 1‐deoxygalactonojirimycin hydrochloride) has first been described as a competitive inhibitor of the α‐Gal A. It has secondly been used at lower dose as an oral pharmacologic chaperone, stabilizing the specific mutant forms of α‐gal A from *amenable* mutations, increasing the enzyme trafficking to lysosomes and therefore the intracellular α‐Gal A activity (Germain et al., 2016). Migalastat has recently been reported to be an effective alternative treatment to ERT in patients with such variants, reducing GL3, stabilizing renal function, reducing cardiac mass, and reducing gastrointestinal disorders (Benjamin et al., 2017, 2009; Germain et al., 2016; Hughes et al., 2017). However, there are few data on the effects of migalastat on α‐Gal activity in vivo. We report a strong increase of leukocyte α‐Gal activity reaching the normal reference range associated with a slight decrease of plasma lyso‐Gb3 and improvement or stabilization of cardiac symptoms in two p.Asn215Ser (p.N215S) Fabry patients treated with migalastat.

## 2 METHODS

Our study was approved by an ethics committee (CPP Ile‐de‐France VI‐3 April 2014; CCTIRS 14.324bis‐18 June 2014; CNIL‐DR‐2014‐506‐28 November 2014). Clinical evaluation, imaging and laboratory tests of two unrelated men referred to our tertiary centers for hereditary cardiomyopathies and FD were performed by the same practitioners and retrospectively reported. Migalastat was administered according to the summary of product characteristics, orally every other day (123 mg). A written consent was received from both patients for collecting and publishing data. Blood samples were collected routinely, between 24 and 48 hr from the last intake of migalastat.

The α‐Gal A activity in blood cells lysate was measured using the fluorogenic substrate 4‐methylumbelliferyl‐α‐D‐galactopyranoside, and normalized to the total protein, as described elsewhere (Daitx et al., 2015). The normal activity in control subjects is ranging from 20 to 60 nmol mg^−1^ hr^−1^ in our laboratory. Leukocyte α‐Gal A activity was measured 1 hr before ERT infusion, and between 24 and 48 hr of the last intake of migalastat. Plasma *lyso*‐Gb3 was measured using liquid chromatography coupled to mass spectrometry, with glycinated *lyso*‐Gb3 as internal standard (normal range <0.8 nmol/L) as previously described by Mauhin et al., (2018). The *GLA* mutations were identified using Sanger sequencing.

## 3 RESULTS

The charts of clinical, biological and morphological data of both cases reported herein are summarized in Table 1.

**Table 1:** Clinical, echocardiographic and biological data of patients at diagnosis, after ERT treatment and after introduction of migalastat

	Patient 1	Patient 2
Year of birth	1967	1963
Treatment lines for FD	Agalsidase beta—6 months (November 2012—May 2013) Agalsidase alfa—19 months (June 2013—February 2015) Migalastat from July 2016	Agalsidase alfa—30 months (December 2014—June 2017) Migalastat from June 2017
Other therapeutics	Candesartan 32 mg/day Aspirine 100 mg/day Melatonine 3 mg/day	Candesartan 8 mg/day Atenolol 50 mg/day Aspirine 160 mg/day
Symptoms at baseline	Anhidrosis—Heat intolerance Hypertrophic cardiomyopathy: conduction defect—pace‐maker (26 years) Hearing loss	Hypertension Hypertrophic cardiomyopathy Hearing loss
Clinical follow‐up under migalastat	Positive: improvement of sudation, heat intolerance, fatigue Negative: flu‐like syndrome (progressively improved in 3 months), headache (progressively improved in 2 months) Rhinitis	Positive : LV mass, good tolerance

	At diagnosis	ERT	Migalastat (9 months after introduction)	At diagnosis	ERT (2.5 years under ERT)	Migalastat (11 months after introduction)
Age (years)	45	45	49	51	51	54
LV Ejection fraction (%)	74	72	84	55	75	74
LV end diastolic diameter (mm)	46.2	46	48	49.5	54.2	56
Thickness of the septum (mm)	13.2	13.7	13.7	16.3	17.2	14.1
Posterior wall thickness (mm)	10.1	13	8.4	14.6	11.3	10.2
Maximal LV wall thickness (mm)	14	14	14	17	19	18
LV mass (g/m²)	98	NA	97	158	152	132
Serum creatinine (µmol/l)	74	59	72	92	93	98
Proteinuria >30 mg/mmol creatinine	No	No	No	No	No	No
a‐GAL A (nmol/h/mg)	6.1	6.1	34	5	10	29
lyso‐Gb3 (nmol/L)	NA	2.3	1.7	NA	3	2.7

Abbreviations: α‐GAL A, leukocyte alpha‐galactosidase A activity; ERT, enzyme replacement therapy; FD, Fabry disease; LV, left ventricle; NA, not available.

### 3.1 Case 1

**Figure 1:**
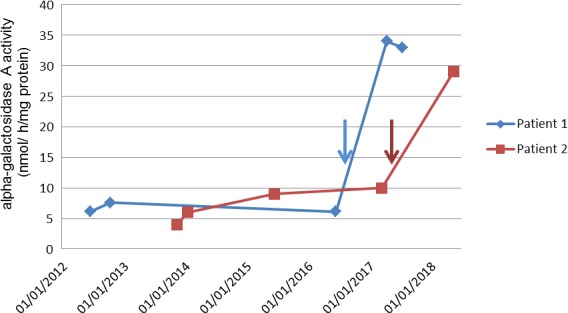
Evolution of leukocyte α‐galactosidase A activity under treatment. (Arrows: introduction of migalastat therapy; brackets: time under enzyme replacement therapy)

A 45‐year‐old man with hypertrophic cardiomyopathy (wall thickness 14 mm) and a history of symptomatic conduction disorders (sinus dysfunction and second‐degree atrioventricular block), who required a pacemaker implantation at 26 years was referred to our cardiologic center in June 2011. FD was diagnosed with a p.N215S variant of GLA. ERT (agalsidase beta) was initiated at the age of 45 and switched to agalsidase alfa after 6 months because of allergic side effects. Two years later, at the age of 49, ERT was replaced with migalastat because of persistent allergic reactions to infusions (skin rashes, arthralgia). The patient reported an important and sustained improvement of his fatigue. Interestingly, the leukocyte α‐Gal A activity was strongly increased, reaching normal ranges after 9 months of treatment (from 6.1 to 34 nmol mg^−1^ hr^−1^). It remained elevated 3 months later (Figure 1), whereas plasma *lyso‐*Gb3 decreased (from 2.3 to 1.7 nM; see Table 1). Echocardiographic parameters were stable (Table 1). Cardiac and brain magnetic resonance imaging (MRI) were not performed because of the pacemaker.

### 3.2 Case 2

A 51‐year‐old man with a history of controlled hypertension and LVMH (17 mm) was diagnosed in 2013 with FD. In his family medical history, his maternal uncle had end‐stage renal disease and died from sudden death at age 49. Molecular analysis revealed the p.N215S variant of GLA. ERT with agalsidase alfa was initiated in 2014. In May 2017, because of the patient's preference for an oral therapy, he switched to migalastat. After 1 year of treatment, the leukocyte α‐Gal A activity normalized, increasing from 5 to 29 nmol mg^−1^ hr^−1^, and *lyso*‐Gb3 plasma level decreased from 3 to 1.5 nM. After 11 months of migalastat, LV mass improved on echocardiography (132 vs. 158 g/m^2^ at baseline; see Table 1). Cardiac MRI remained stable for LV wall thickness, late enhancement in infero‐latero‐basal segments and native T1 mapping relaxation time (865 ms at baseline). NT‐proBNP level was stable (99 ng/ml vs. 107 ng/ml at baseline).

## 4 DISCUSSION

We report a normalization of leukocyte α‐Gal A activity (fivefold increase) in two FD patients with chaperone therapy (migalastat). The two unrelated patients harbored the same p.N215S variant of the exon 5 in GLA that has been associated with the late‐onset cardiac phenotype of FD (Oder et al., 2017). Nevertheless, p.N215S carriers can be affected by the classical systemic phenotype of FD, as did the relative of patient 2 (Germain et al., 2018).

The benefits of ERT have been widely described but some situations can limit its use such as the development of an immune reaction against ERT or the necessity of biweekly IV infusions. Moreover, ERT has been shown to be less effective in patients with advanced stage of LV or renal fibrosis (Weidemann et al., 2013). Migalastat has been shown to be biochemically effective in vitro by chaperoning *amenable* mutated α‐Gal A to lysosomes, mimicking natural enzyme trafficking, and increasing α‐Gal A activity (Benjamin et al., 2009). Amenability of each genetic variant has been assessed with an in vitro assay in which each of the 600 primarily missense FD–causing mutations were expressed in a human embryonic kidney 293 cell‐line for testing and measuring increases in α‐Gal A activity in response to migalastat. An *amenable* mutant form is defined by an increase of α‐Gal A activity induced by migalastat (Benjamin et al., 2017). In a phase 2 study, the coadministration of migalastat and agalsidase resulted in a 2 to 4.5‐fold increase in α‐Gal A plasmatic activity compared to agalsidase administered alone (Warnock et al., 2015). To date this association has not been validated by the FDA or EMA, in part due to the high cost of both treatments. Interestingly, the best performances were observed in FD patients’ fibroblasts with p.N215S mutation (Benjamin et al., 2009).

A recent study showed a significant increase of leukocyte α‐Gal A activity, but not a complete normalization, in 14 FD patients (three females) after 1 year of migalastat, increasing from 0.06 (IQR 0.04–0.12) to 0.2 nmol min^−1^ mg^−1^ protein (IQR 0.06–0.26; reference values reported in the publication: 0.4–1.0 nmol min^−1^ mg^−1^ protein) (Müntze et al., 2019). In this study, the subgroup of p.N215S carriers (*n* = 8), had an increase of α‐Gal A activity from 0.06 (0.05–0.07) to 0.2 nmol min^−1^ mg^−1^ protein (0.1–0.25) (Müntze et al., 2019). Although blood leukocytes are not the main therapeutic target in FD, intracellular leukocyte α‐Gal A activity reflects the amount of enzyme that could reach the lysosome via intracellular trafficking. In contrast with our observation, leukocyte α‐Gal A activities increased to a lesser extent in this previous paper. To explain the difference we observed, we could hypothesize several explanations including a better absorption of migalastat in our patients. The efficacy of migalastat depends on the patient's diet and on the timing of the drug administration, with a 40% decreased concentration if administered 1 hr around the meal (Johnson, Mudd, & Janmohamed, 2015). Also we systematically propose the use of a smartphone application to improve the adherence*.* While the half‐life of migalastat is about 3.2–4 hr, the time of sampling from the drug intake could also be discussed to explain the difference of enzyme activity (Ino, Takahashi, Terao, Mudd, & Hirama, 2013). However, we did not observe differences in dosages performed between 24 and 48 hr of the last intake. We could not assess the role of possible genetic polymorphisms in these better responses to migalastat. Our observation is obviously limited by the number of patients and may be restricted to the p.N215S genotype. However, this mutation is one of the most prevalent (Eng, Resnick‐Silverman, Niehaus, Astrin, & Desnick, 1993). Our observation needs to be confirmed in larger cohorts including p.N215S patients, as well as patients with renal involvement.

In conclusion, we report the normalization of leukocyte α‐Gal A activity in FD patients treated with migalastat, in contrast with conventional ERT. This finding illustrates the benefit of a personalized treatment of inheritable metabolic diseases. The correction of α‐Gal A activity and the tolerance of the drug suggest that migalastat may be a treatment of choice in FD patients with *amenable* mutations, especially in p.N215S patients.

## CONFLICT OF INTERESTS

FL has received travel support from Amicus Therapeutics, Shire, and Sanofi‐Genzyme. He received lecture fees from Actelion Pharmaceuticals. WM has received travel support from Amicus Therapeutics, Shire, Orphan Europe, and Sanofi‐Genzyme. He received lecture fees from Shire and Amicus Therapeutics. FK, WK, CB and JL declare no conflict of interest. OL has received travel support and lecture fees from Amicus Therapeutics, Shire, and Sanofi‐Genzyme. PC has received travel support and/or lecture/consulting fees from Amicus Therapeutics, Shire, and Sanofi‐Genzyme. He also received research grant from Shire.
